# Difference in glaucoma progression between the first and second eye after consecutive bilateral glaucoma surgery in patients with bilateral uveitic glaucoma

**DOI:** 10.1007/s00417-016-3460-5

**Published:** 2016-08-06

**Authors:** Norshamsiah Md Din, Lazha Talat, Hazlita Isa, Oren Tomkins-Netzer, Keith Barton, Sue Lightman

**Affiliations:** 1 UCL Institute of Ophthalmology, 11-43 Bath Street, London, EC1V 9EL UK; 2 UCL Institute of Ophthalmology, Moorfields Eye Hospital, City Road, London, EC1V 2PD UK; 3 Universiti Kebangsaan Malaysia, Jalan Yaacob Latiff, Cheras, 56000 Kuala Lumpur, Malaysia; 4 NIHR Biomedical Research Centre for Ophthalmology, Moorfields Eye Hospital, London, UK

**Keywords:** Uveitic glaucoma, Glaucoma progression, Filtering surgery, Progressor software

## Abstract

**Purpose:**

To determine whether the second eyes (SE) of patients with bilateral uveitic glaucoma undergoing filtration surgery have more glaucomatous progression in terms of visual acuity, visual field (VF) and optic nerve changes compared to the first eyes (FE).

**Methods:**

This retrospective study analysed data of 60 eyes from 30 patients with bilateral uveitic glaucoma who had undergone glaucoma surgery in both eyes on separate occasions. Humphrey VF progression was assessed using the Progressor software.

**Results:**

The pre-operative IOP between the FE (43.1 ± 7.7 mmHg) and SE (40 ± 8.7 mmHg) was not statistically significant (*p* = 0.15). IOP reduction was greater in the FE (64 %) than SE (59.7 %) post-operatively, but the mean IOP at the final visit in the FE (12.3 ± 3.9 mmHg) and SE (14.5 ± 7 mmHg) was not statistically different (*p* = 0.2). There was no significant change in mean logMAR readings pre and post-operatively (0.45 ± 0.6 vs 0.37 ± 0.6, *p* = 0.4) or between the FE and SE. The number of SE with CDR > 0.7 increased by 23 % compared to the FE. From 23 available VFs, five SE (21.7 %) progressed at a median of five locations (range 1–11 points) with a mean local slope reduction of 1.74 ± 0.45 dB/year (range −2.39 to −1.26), whereas only one FE progressed. However, there was no significant difference between mean global rate of progression between the FE (−0.9 ± 1.6 dB/year) and SE (−0.76 ± 2.1 dB/year, *p* = 0.17) in the Humphrey VF.

**Conclusion:**

In eyes with bilateral uveitic glaucoma requiring glaucoma surgery, the SEs had more progressed points on VF and glaucomatous disc progression compared to FEs at the final visit.

## Introduction

Uveitis is bilateral in approximately 75 % of patients, and about 40 % suffer bilateral intraocular pressure (IOP) elevation at some point in the course of the disease [1–3]. These patients often have chronic uveitis and poor vision if left untreated [4]. Topical hypotensive agents are able to control the IOP in 26 % of patients and an additional 62 % required oral carbonic anhydrase inhibitors (CAI) such as acetazolamide for additional IOP control [5]. Despite that, about 35 % of adults and 60 % of children with uveitic glaucoma inevitably need glaucoma surgery, either because of oral CAI dependency or insufficient IOP control while on maximum tolerated medical therapy (MTMT) [5]. IOP can be severely elevated and insufficiently controlled with medical therapy, and therefore requires early surgical intervention for IOP control even in the absence of glaucomatous disc changes [6].

Glaucoma assessment takes into account progression of the optic disc cupping [7], visual field (VF) [8], peripapillary nerve bundle atrophy [9] and more recently, retinal nerve fiber layer thickness [10, 11]. Visual field progression has been derived almost exclusively from standard automated perimetry (SAP), and many studies have used only SAP as a primary functional endpoint [12–14]. The Humphrey VF is one of the most widely performed tests for diagnosis and monitoring of glaucoma progression. Progression can be assessed either by event-based analysis (i.e., whether VF progression has occurred or not) [15] or trend analysis of the rate of disease progression [12–14]. Trend analysis with point-wise linear progression (PLR) uses the rate of loss in decibels per year (dB/year) to describe localized points that are actively progressing.

In most patients, systemic CAI is stopped in the early post-operative period to prevent post-operative hypotony and ensure maintained aqueous flow through the bleb. With this, the second eye (SE), which may also be dependent on oral CAI for IOP control, could develop marked IOP elevation. Depending on the severity of IOP elevation and the interval between the first and second surgery, the SE may suffer glaucoma progression more than the first eye (FE). This study was therefore performed to determine whether this is in fact the case in terms of increased cup to disc ratio (CDR), VF progression, and worsening of visual acuity.

## Materials and methods

A retrospective chart review was conducted among patients who attended the uveitis clinic in Moorfields Eye Hospital from May 2010 to Nov 2012. Patients with bilateral uveitis who had undergone bilateral glaucoma surgery, either trabeculectomy or aqueous shunt device, with or without antimetabolite augmentation, and had been followed up for up to 5 years after surgery were included. Exclusion criteria were patients with missing data or inadequate follow-up. The Research Governance Committee of Moorfields Eye Hospital approved the data collection (protocol LIGS 10201). The study adhered to the tenets of the Declaration of Helsinki.

The pre-operative data refers to the point when the decision for surgery was made, i.e., when the patients were on MTMT. Data collected included age, anatomical uveitis type, glaucoma treatment, vertical CDR as reported in the case notes, the best-corrected visual acuity (BCVA) pre-operatively and annually until 5 years after their respective surgeries, the highest IOP reading recorded during the entire period before and after surgery (excluding any IOP elevation within 2 months post-operatively to account for post-surgical IOP fluctuation) [16], the pre-operative IOP prior to their respective surgeries and annually until 5 years post-operatively, the status of the lens at the time of surgery (either phakic, pseudophakic, or aphakic), and Humphrey VF data.

The amount of IOP reduction after surgery was described as the percentage of IOP reduction from the pre-operative IOP and at 1 year post-operatively. IOP readings were taken using the Goldmann Applanation Tonometer. The visual acuity was tested with the Snellen chart using the patients’ spectacles if worn and then retested with the addition of a pinhole. The best vision achieved (BCVA) was recorded. Impaired vision was defined as BCVA between 6/15 and better than 6/60, whereas poor vision was defined as BCVA poorer than 6/60 according to the SUN classification [17]. When comparing pre- and post-operative BCVA, the logarithm of the reciprocal of the decimal BCVA was used to approximate the logMAR. Eyes without form vision were classified into one of the low-vision categories of logMAR conversion: counting fingers = 2.0 and hand motions = 2.3 [18]. Light perception and no light perception were excluded, as they were considered not a formed perception and could not be quantified [19].

Surgical successes were defined as IOP of ≤21 mmHg at 1 year post-operatively, either with glaucoma medications (qualified success) or without glaucoma medications (complete success) [20, 21]. Post-operative hypotony was defined as IOP <6 mmHg on two consecutive visits, or one visit requiring intervention. An eye with a vertical CDR of more than 0.7 or reported as having glaucomatous changes such as focal notching, rim pallor, or excavation of the rim is considered to have glaucomatous disc changes [22].

VFs were done every 4–6 months, depending on the necessity as judged by the attending ophthalmologist. Progression of glaucomatous VF loss was assessed using the Progressor software (version 3.3; Medisoft Inc, London, United Kingdom). The software calculates point-wise linear regression (PLR) analysis and provides local slopes of progression (in dB/year) for each of the 59 locations in the VF as well as globally, and its level of significance (*p* values). A Gaussian filter was applied to reduce measurement variability without additional testing or exclusion of noisy tests to allow inclusion of all available VF tests irrespective of reliability criteria [23]. A test point was considered progressing if the slope of sensitivity over time exceeded 1 dB/year (with *p* < 0.01). For edge points, a stricter slope criterion of >2 dB loss/year (also with *p* < 0.01) was used [24].

Data were analysed using Stata version 10 (Intercooled) (StataCorp, College Station, TX, USA). All categorical variables were compared using chi-square test and a paired *t*-test was used to compare means for normally distributed data. The Mann–Whitney test was used to compare means of non-parametric data. For data counts of less than 5 in any subgroups, the Fisher exact test was used to compare proportions. Survival analysis and Kaplan–Meier graphs were plotted to assess the annual probability of surgical success in the first 5 years post-operatively. A linear mixed model analysis was used to assess the annual rate of visual loss and progression of the vertical CDR in the first 5 years post-operatively. Cox’s regression analysis was performed to assess the risk factors for surgical failure, and multivariate logistic regression analysis was used to determine the risk factors for bleb failure in trabeculectomy. Results are reported as mean and standard deviation unless stated otherwise.

## Results

A total of 43 patients with uveitis underwent bilateral glaucoma surgery. Thirteen patients were excluded because of missing data (*n* = 9), inadequate follow-up period (*n* = 3) and co-existence of retinitis pigmentosa (*n* = 1). Therefore, 60 eyes of 30 patients were included in the analysis.

The male to female ratio was 1:1.3. The mean age was 39 years (range 7–66 years), with seven children and 23 adults. The FE was the right in 16 patients and left in 14 patients. Fourteen patients (46.7 %) had anterior uveitis, eight patients (26.7 %) had intermediate uveitis, and a further eight patients (26.7 %) had posterior/panuveitis. The mean follow-up period was 12.0 ± 9.4 years (range 3–42 years).

Glaucoma surgery was performed on the FE at a median duration of 6.5 years (range 1–36 years) after the diagnosis of uveitis. The first operated eye was the eye with more poorly controlled IOP. The timing for the SE surgery depended on the IOP control while on medical treatment. In SEs with poor IOP control, or borderline IOP control with a glaucomatous disc, surgery is performed earlier. The mean interval between surgery of the FE and SE was 30.3 months (range: 0.25 to 180 months). Nine SEs (30 %) had surgery within 6 months after the FE surgery, and 21 SEs (70 %) had it after 6 months. Forty-one eyes (68.3 %) had trabeculectomy, and 19 eyes (31.7 %) had aqueous shunt implantation (three Molteno and 16 Baerveldt devices) with or without antimetabolite augmentation.

When operating the FE, ten of 30 SE (33 %) had no glaucomatous disc changes, with a mean IOP of 32 and 30 mmHg in the FE and SE respectively. Some of the SEs were controlled medically until the decision for the SE surgery was made. Both FE and SE received approximately the same mean number of drops (3.2 and 3.1 in the FE and SE respectively).

### Pre-operative treatment and the indications for surgery

The mean number of pre-operative topical classes of glaucoma medications was 3.1 ± 0.88 drops. Twenty-seven eyes (45 %) had four medications, 21 eyes (35 %) had three medications, nine eyes (15 %) had two medications, and three eyes (5 %) had one type of medication pre-operatively. Patients who had less than four medications could not tolerate other medications due to their side-effects. Twenty-one of 30 patients (70 %) received oral acetazolamide before surgery of the FE, which was stopped immediately after the FE surgery.

We compared the highest pre-operative IOP in the FE and SE to determine whether cessation of oral acetazolamide had led to severe IOP elevation in the SE. We found no statistically significant difference in the mean highest pre-operative IOP between the FE (43.2 ± 7.8 mmHg, range 28–56 mmHg) and SE (39.8 ± 8.7 mmHg, range 26–65 mmHg), *p* = 0.15. The indications for glaucoma surgery were: (1) dependency on oral acetazolamide in nine eyes (15 %), four of them SE, (2) advanced glaucomatous disc cupping at borderline IOP control (IOP 19–21 mmHg) on MTMT in 11 eyes (18.3 %), and (3) insufficient IOP control on MTMT with or without glaucomatous cupping in the remaining 40 eyes (66.7 %). There was no difference in the distribution of FE and SEs according to IOP, CDR, and number of glaucoma drops pre-operatively (Table 1).

**Table 1:** Comparison of study outcomes between the first and second eye before surgery and at the final visit

Study outcome	Before surgery			At final visit		
	FE, *n* = 30	SE, *n* = 30	*P*	FE, *n* = 30	SE, *n* = 30	*P*
BCVA in logMAR, median	0.3	0.2	0.43^a^	0.2	0.3	0.47^a^
BCVA, no. of eyes (%)						
≥6/15	8 (26.7)	6 (20.0)		4 (13.3)	13(43.3)	
≥ 6/60	1 (3.3)	4 (13.3)	0.47^b^	5 (16.7)	1 (3.3)	0.02^b^
CDR ≥0.7, no. of eyes (%)	19 (65.5)	13 (44.83)	0.12^c^	19 (65.52)	16 (55.2)	0.42^c^
CDR, mean (SD)	0.67 (0.24)	0.54 (0.25)	0.06^a^	0.67	0.63	0.4
IOP^e^, mean (SD)	35.2 (12.2)	31.4 (9.4)	0.2^d^	12.3 ± 4	14.5 ± 7	0.2^d^
Number of classes of glaucoma drugs, mean (SD)	3.23 (0.9)	3.1 (0.9)	0.6^a^	1.8	2.3	0.3^a^

*BCVA* best-corrected visual acuity, *FE* first eye, *SE* second eye, *CDR* cup to disc ratio, *SD* standard deviation, *IOP* intraocular pressure

^a^Mann–Whitney test

^b^Fisher’s exact test

^c^Chi square test

^d^paired *t*-test

^e^the IOP before surgery refers to the IOP on maximum tolerated medical therapy

**Fig. 1:**
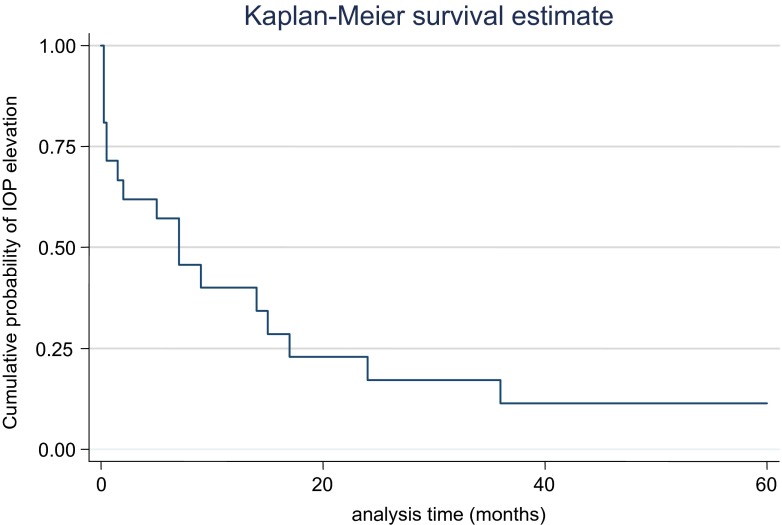
Kaplan–Meier graph illustrating the cumulative probability of normal IOP in the second eye after oral acetazolamide was stopped following surgery in the first eye. IOP elevation was defined as IOP > 22 mmHg

The Kaplan–Meier graph in Fig. 1 illustrates the cumulative probability of normal IOP in the SE after cessation of oral acetazolamide following the first surgery against time. IOP elevation was observed in 43 % of SEs within the first year after the FE surgery, 18.4 % in the second year, and 12.2 % in the third, fourth, and fifth year respectively.

### Post-operative outcome

#### Post-operative outcome in patients who received oral acetazolamide before the FE surgery

Out of 21 patients who received acetazolamide, seven SEs (16.7 %) vs four FEs (9.5 %) showed progression in CDR, while four SEs (9.5 %) and two FEs (4.7 %) had VF progression after a mean duration of 9 ± 5.7 years postoperatively. The mean pre-operative highest IOP in the SEs (36 ± 8.7 mmHg) was also higher than the FE s(32 ± 7.8 mmHg) although it did not reach statistical significance (*p* = 0.06).

In SEs which had surgery > 6 months after FE surgery, IOP in the SE could be controlled medically for the first few months. Of the 21 SEs which had surgery > 6 months after FE surgery, three eyes (14 %) had disc progression and four eyes (19 %) had VF progression, as opposed to one of the nine SEs (11 %) which had surgery < 6 months after FE surgery developed disc and VF progression. However, because of the small number of eyes affected, statistical significance was not achieved.

#### Visual acuity

While there was no eye with light perception vision pre-operatively, two FEs had final VA of light perception. The underlying causes were advanced GON alone and a dense cataract with advanced GON in each eye.

To estimate the mean rate of change in BCVA within the first 5 postoperative years, a linear mixed model of mean logMAR was fitted, with the postoperative years as the independent variable and the FE and SE nested within patients. The estimated mean rate of progression was 0.02 logMAR per year and this was not found to be statistically significant (coefficient estimate 0.02, *p* = 0.38, 95 % CI −0.026 to 0.07). The estimated mean rate of progression in the FE was 0.05 logMAR per year (coefficient estimate 0.052, *p* = 0.143, 95 % CI: −0.018 to 0.122) and in the SE was −0.02 logMAR per year (coefficient estimate −0.02, *p* = 0.473, 95 %CI: −0.076 to 0.035).

In general, there was neither a significant difference in the median logMAR BCVA pre-operatively (0.3) and at the final visit (0.2, *p* = 0.4), nor was there any significant difference in the distribution of FEs and SEs with impaired and poor vision pre-operatively. However, at the final visit, there were significantly more FEs with poor vision and more SEs with impaired vision, *p* = 0.02 (Table 1).

Of the eyes with final BCVA poorer than 6/60 (*n* = 6), in only one eye was this exclusively due to advanced GON. The rest was due to hypotony maculopathy following glaucoma surgery and related to low IOP, choroidal neovascular membrane at the macula, combination of aphakia, band keratopathy and advanced GON, and a dense cataract (*n* = 1 respectively).

#### Optic disc appearance

Two eyes were excluded in the analysis of optic disc appearance because the discs were obscured by a dense cataract and band keratopathy (one eye each) at the final visit. Among eyes where the optic disc was visible (*n* = 58), 32 eyes (55.2 %) had CDR > 0.7 pre-operatively. This number increased by three eyes (9.4 % increment) at the last visit, and this increase occurred in the SEs (3/13 eyes, 23.1 % increment). A linear mixed model analysis revealed no significant CDR progression between the FEs and SEs within the first 5 years post-operatively (mean CDR 0.6, coefficient estimate 0.006, *p* = 0.443, 95 % CI −0.01 to 0.02).

**Fig. 2:**
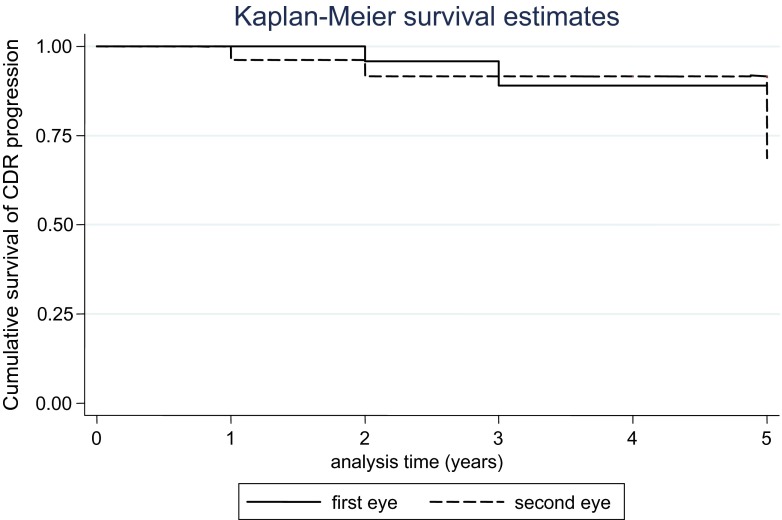
Kaplan–Meier graph showing the difference in cumulative survival risk of CDR progression between the first eye (*FE*) and second eye (*SE*)

To cater for variability in reporting CDR between observers in the clinic, progression of the CDR was considered present when the ratio was reported as increasing by 0.2 or more. With this definition, five eyes progressed within 5 years post-operatively, which were 2 FEs and 3 SEs. Although it appeared from the Kaplan–Meier graph in Fig. 2 that the rate of CDR progression is higher in the SEs, a log rank test revealed no significant difference in the overall CDR progression between the FEs and SEs, *p* = 0.34. The survival rates of CDR progression in the FEs and SEs at 1, 2, and 5 years were 100 and 96.1 %, 95.8 and 91.6 %, 89 and 68.7 % respectively (Fig. 2).

#### Intraocular pressure, IOP-lowering treatment and surgical success

The mean IOP in all eyes reduced from 33.2 ± 10.7 mmHg pre-operatively to 14.0 ± 4.7 mmHg at 1 year post-operatively, resulting in a mean decline of 19.5 mmHg after 1 year (57.8 % reduction, *p* < 0.001). This reduction was more in the FE (from a mean of 35.2 ± 12.2 to 12.6 ± 4.5 mmHg, 64.2 % reduction), than the SE (from a mean of 31.4 ± 9.2 to 15.25 ± 7.8 mmHg, 51.4 % reduction) at 1 year post-operatively. However, the mean IOP in the FE and SE at 1 year postoperatively (*p* = 0.23) and at the final visit (12.7 ± 5.4 vs 14.1 ± 7.0 mmHg in the FE and SE respectively, *p* = 0.4) was not statistically significant.

**Fig. 3:**
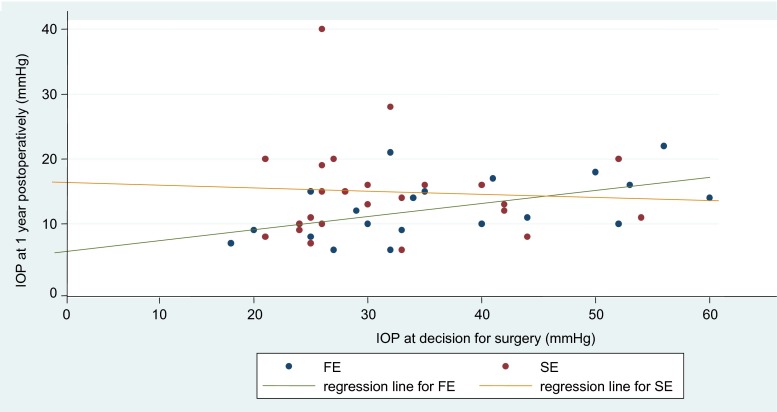
Distribution of the IOP at the decision for surgery and at 1 year postoperatively in the first and second eye. *IOP* = Intraocular pressure, *FE* = first eye, *SE*= second eye

Figure 3 demonstrates the distribution of pre- and post-operative IOP in the FE and SE. The bisecting line showed that the IOP was lower after surgery in the FE but was not significantly so in the SE. The regression line for the FE showed that a higher pre-operative IOP is associated with a higher IOP at 1 year post-op (correlation coefficient, *r* = 0.53, *p* = 0.01), whereas this positive correlation was not seen in the SE (correlation coefficient, *r* = −0.06, *p* = 0.78).

**Fig. 4:**
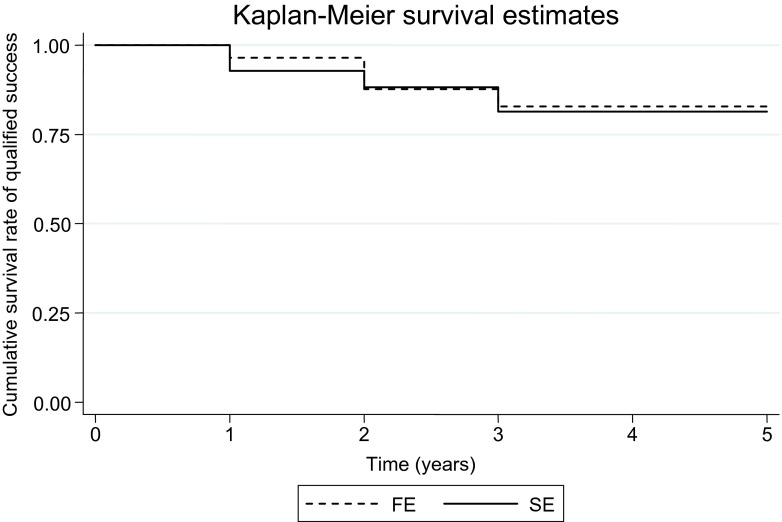
Kaplan–Meier graph comparing the cumulative survival rate of qualified success between the first eye (*FE*) and second eye (*SE*)

The overall qualified success at 1 year post-operatively was 92.5 %, and complete success was 73 %. There was no significant difference in the survival rates of qualified success between the FE and SE, *p* = 0.86. The survival rates of qualified success of the FE and SE were 96.4 and 93 % at 1 year, 87.6 and 88.2 % at 2 years, 83 and 81 % at 5 years postoperatively (Fig. 4). There was also no significant difference in the survival rate between SEs that had surgery within 6 months or after 6 months post FE surgery. In eyes with equally high IOP before the first surgery, bilateral surgery was planned to be performed within a short period between the two eyes.

Cox regression analysis was performed to evaluate the risk factors for surgical failure (IOP >21 mmHg) within the first 5 post-operative years. Factors included were the patients’ age at the time of surgery, the number of pre-operative glaucoma medications, and the state of the lens at the time of surgery. The type of glaucoma surgery (whether trabeculectomy or aqueous shunt) was not a significant factor (Hazard ratio, HR 1.3, *p* = 0.7) and therefore was not included. After adjusting for the other factors, the risk of failure reduced by 37.7 % with every 10-year increase in age (adjusted HR = 0.623, *p* = 0.04, 95 % CI: 0.39–0.99). The number of pre-operative glaucoma drops and the state of the lens at the time of surgery was not a significant factor for surgical failure in this model.

At the final visit, 34 eyes (53.1 %) had IOP ≤ 12 mmHg and four eyes (6.25 %) had IOP ≥ 21 mmHg. A total of 28 eyes (43.7 %) were on hypotensive agents, six eyes were on one, seven eyes on two, ten eyes on three, and five eyes on four agents. Twelve out of 41 trabeculectomy eyes (29.3 %) required a secondary procedure, either a revision of trabeculectomy, needling, or implantation of aqueous shunt with or without 5-Fluorouracil. Hypotony occurred in 12 eyes (18.7 %), intravitreous haemorrhage occurred in one eye, and another eye had to undergo reformation of anterior chamber with viscoelastic agent. A multivariate logistic regression analysis revealed that aphakic or pseudophakic eyes undergoing trabeculectomy were 4 times more likely to undergo a secondary procedure compared to phakic eyes after adjusting for age (OR 4.5, *p* = 0.04, 95 % confidence interval 1.1–19.6). The number of pre-operative glaucoma drops was not found to be a significant risk factor, and was therefore not included in this model.

#### Visual field analysis

Of the 30 patients, 23 had sufficient VF tests for analysis, with a mean of 6.5 tests per patient (range: three to 15 tests). Ten of 36 eyes (28 %) with VFs had less than five VF tests performed. This was because some eyes had very poor vision to perform a meaningful VF, and the decision for surgery has to be made based on IOP control and disc changes. Overall, there was no significant difference between the mean number of progressed points pre-operatively (0.17 points, range 0–3) and at the final visit (0.63 points, range 0–11), *p* = 0.28. The SE showed progression in five patients (21.7 %), the FE progressed in one patient (4.3 %), and no progression occurred in either eye in the remaining 17 patients (74 %) at the final visit. In the SEs that progressed, the median number of progressed points was five locations (range 1 to 11 points), with a mean local slope reduction of −1.74 ± 0.45 dB/year (range −2.39 to −1.26). There was no significant difference in the mean reduction of the global slope between the FE and SE postoperatively (−0.75 ± 0.6 and −0.9 ± 0.4 dB/year respectively, *p* = 0.83).

A multivariate linear regression was performed to determine the risk factors for local and global slope progression. The factors examined were the occurrence of hypotony, the highest IOP reading after surgery, the time from the first to second surgery, and the duration of follow-up. We found no association between the local and global slope progression with the risk factors, possibly because of the small number of eyes that progressed.

However, when examining the risk factors for increased number of progressed points, hypotony was found to be associated with increased number of progressed points by 0.7 points (regression coefficient 0.68, 95 % CI 0.01–1.34, *p* = 0.046) after adjusting for the highest IOP and duration of follow-up.

## Discussion

This study was performed to answer the question of whether cessation of oral acetazolamide following glaucoma surgery of the FE may be associated with severe IOP elevation and hence more glaucoma progression in the SE compared to the FE. It involved relatively young patients (mean age 39 ± 22.1 years) who had developed elevated IOP as a complication of chronic uveitis or corticosteroid treatment. There was a wide range of follow-up, which may influence some of the progression seen in these patients.

In 70 % of SEs, raised IOP requiring glaucoma surgery occurred 6 months or later after the first surgery, suggesting that majority of the second surgery can be delayed until the FE has stabilized. Cessation of acetazolamide did not result in a higher pre-operative maximum IOP in the SE compared to the FE. This might indicate that with acetazolamide withdrawal following the first surgery, the SE IOP did not go higher but reverted back to the level before the FE surgery.

Our mean pre-operative IOP while on MTMT (33.2 mmHg) was comparable to the 33.7 mmHg mean pre-operative IOP in a study on long-term outcome of trabeculectomy in 101 uveitic eyes [16], although our patients had more pre-operative drops (3.1 vs 2.86 drops), and more patients were prescribed oral acetazolamide (70 % vs 66.3 %) when compared to those in their study. This could indicate that medical treatment may only reduce the IOP up to a certain level and that surgery in this group of patients is inevitable. Nearly 67 % of eyes in our study had insufficient IOP control (IOP >21 mmHg) prior to surgery whilst on MTMT, comparable to the 60 % reported success of non-surgical treatment in children with uveitis [5].

Unlike primary glaucomas, surgery is performed in uveitis for IOP control, even in the absence of glaucomatous optic neuropathy [6]. This is because the IOP may continue to increase to an unacceptable level despite MTMT because of persistent steroid therapy required to maintain uveitis inactivity. Nevertheless, progressive glaucomatous damage could be seen post-operatively, more in the SE than the FE, and occurring both anatomically (increase in eyes with CDR > 0.7) and functionally (increase in number of progressed points on VF test).

In general, glaucoma surgery in our study resulted in a mean IOP reduction by 57.8 %, slightly lower than that reported in other studies involving uveitic eyes (59 %) [21] but higher in those of non-uveitic eyes (40 %) [13]. However, this difference is due to the different definitions of pre-operative IOP used. The first study used the highest IOP reading, whereas the second study took the average of all IOP readings pre-operatively. We defined pre-operative IOP as the IOP while on MTMT to illustrate that any further IOP reduction following surgery was from the effect of surgery alone and not from any contribution from medical therapy. Although mean IOP reduction was more in the FE (64.2 %) than SE (51.4 %), the mean IOP at 1 year post-operatively and at the final visit was not statistically different between the two eyes. The difference in the period between the first and second surgery did not have any effect on the surgical success in the SE (Fig. 4). Furthermore, the difference with regard to VF and CDR changes in the FE and SE is only moderate considering that the delay between the FE and SE surgery is quite long.

Although greater IOP reduction can often be achieved with surgery when medical therapy is insufficient, surgical intervention may not be enough to cease VF progression, as IOP fluctuation has also been identified as a risk factor for VF progression, especially in advanced glaucoma and low mean IOP [25–27]. Postoperative hypotony occurred in 16 % of our patients (20 % of eyes), slightly lower than the figure quoted by previous studies on uveitic eyes (19.4 %) [21]. A multivariate linear regression was unable to demonstrate any association between the global and local slope progression of the VF with either the occurrence of hypotony or the maximum IOP reading after surgery, probably because of the small number of eyes that progressed. However, hypotony was the only significant risk factor found to be associated with an increased number of progressing points.

Qualified success in our study at 1 year post-operatively was 92.5 % and complete success was 73 %, comparable to a study by Chawla et al. who reported 90.3 and 71 % of qualified and complete success respectively at 1 year post trabeculectomy on adult uveitic patients [21]. Although there was no difference in the survival rates of qualified success between the FE and SE, the rates steadily decreased in both eyes within the first 5 years. As a result, glaucoma progression can be seen as a steady decrease in the survival rates of CDR progression, more in the SE, although this does not translate into significant VF progression both in the local and global slope of the point-wise linear regression.

While the use of multiple hypotensive agents pre-operatively and the state of the lens (aphakia/pseudophakia) were found to be responsible for a higher failure rate in non-uveitic glaucoma surgery [27], we found no association between them and the odds of surgical success in uveitis. It could be that success of glaucoma surgery in uveitis is influenced by many other factors different to those in non-uveitic glaucomas. On the other hand, we found younger patients more likely to develop surgical failure, agreeing with previous studies [28], possibly because of their inclination to mount scar tissue formation compared to the elderly. However, because surgical success only takes into account the IOP at any given time, other bleb-saving procedures were not demonstrated as surgical failures.

The drawbacks of VF progression analysis in our cohort of patients were that the number and interval of VF between patients were unequal, which may fail to demonstrate the true VF progression. Analysis with the Progressor software in our study allowed for inclusion of all available VFs by using the Gaussian filter. Even so, we found no significant difference in the mean number of progressed points pre-operatively and at the final visit. Additionally, there were no progressed points in about three quarter of patients. In eyes that did progress, the global rate of progression was more in the SE than the FE post-operatively albeit an insignificant difference. The SE progressed in five patients, as opposed to one patient with FE progression. The mean number of progressed points in our study (0.63 points) was far more from the one reported by Fulgar et al. (0.07 progressed points) in a study involving 28 patients with a mixture of POAG, angle-closure glaucoma, and pseduexfoliation glaucoma [13]. Again, this higher figure of progressed VF points in our cohort of patients could be attributed to other pathologies related to uveitis, such as retinal and macular scars, apart from the severity of GON. Even if we have achieved comparable IOP reduction with previous studies, our patients still eventually lose vision.

Consistent with recommendation by previous reports, trabeculectomy was the primary surgical procedure in nearly 70 % of eyes in our study. Traditionally, trabeculectomy with an antifibrotic agents has been the initial procedure in patients with glaucoma who have failed medical and/or laser therapy, and aqueous shunt only comes into play when trabeculectomy fails or in the presence of other factors such as aphakic and pseudophakic patients, JIA-related uveitis, or other risk factors for trabeculectomy failure [6, 29–31]. Therefore, the success rate of aqueous shunt would be expected to be limited because of the refractory nature of the patients’ eyes following one or more failed filtering surgeries. However, there has been increasing evidence in recent years that aqueous shunt may be the initial procedure of choice over trabeculectomy because of the dreaded complications of trabeculectomy such as bleb-related infections, bleb leaks and bleb dysthesia [32]. Some studies have reported comparable success rate of trabeculectomy with mitomycin C in uveitic glaucoma and POAG [33]. Almost 30 % of trabeculectomized eyes in our study required a secondary procedure, either revision of trabeculectomy or aqueous shunt implantation after a failed trabeculectomy.

The limitations of this study are mainly because of its retrospective design and relatively small sample size, although large for the type of patients. Estimation of the CDR is highly variable between different observers in the clinic; and optic disc stereo-photograph, which is the gold standard for estimating progression, was not available in all patients. VFs were also performed at irregular intervals, making standardization of VF progression difficult. However, the findings of this study are still relevant to illustrate the clinical outcome in patients with bilateral uveitis and raised IOP. This group of patients with recalcitrant IOP elevation does show glaucoma progression in certain aspects, even though sufficient IOP control was attained. The SE scored lower in a few aspects, namely structurally (more SEs with CDR > 0.7 at the final visit) and to a lesser extent, functionally (more progressed points and higher progression rate in those points). But because of its small sample size and wide range of follow-up duration, statistical significance was not achieved. A well-designed long-term prospective study with sequential optic disc photographs would be able to determine optic disc progression better. The findings in this study may suggest that in some eyes, earlier glaucoma surgery may probably improve the prognosis of the second eye.

In conclusion, the SE scored lower than the FE as there were more SEs with impaired vision, more progressed points on Humphrey VF, and more eyes with CDR ≥ 0.7 at the final visit. This is especially so in patients who have received oral acetazolamide before the FE surgery. Cessation of oral acetazolamide causes glaucomatous progression in approximately double the number of SEs compared to the FEs, although pre-operative IOP was not significantly different between the two groups of eyes. This is clinically significant, as the final visual outcome may be influenced by both glaucoma and uveitis activity. However, there was no difference in the final logMAR BCVA, the final IOP, and the final global progression on VF. Even if we have achieved comparable IOP reduction with previous studies, our patients still eventually lose vision from glaucoma and the effects of uveitis.

## References

[1] Sijssens KM, Rothova A, Berendschot TTJM, de Boer JH (2006). Ocular hypertension and secondary glaucoma in children with uveitis. Ophthalmology.

[2] Kump LI, Cervantes-Castañeda RA, Androudi SN, Foster CS (2005). Analysis of pediatric uveitis cases at a tertiary referral center. Ophthalmology.

[3] BenEzra D, Wysenbeek YS, Cohen E (1997). Increased intraocular pressure during treatment for chronic uveitis. Graefes Arch Clin Exp Ophthalmol.

[4] Cabral DA, Petty RE, Malleson PN, Ensworth S, McCormick AQ, Shroeder ML (1994). Visual prognosis in children with chronic anterior uveitis and arthritis. J Rheumatol.

[5] Heinz C, Koch JM, Zurek-Imhoff B, Heiligenhaus A (2009). Prevalence of uveitic secondary glaucoma and success of nonsurgical treatment in adults and children in a tertiary referral center. Ocul Immunol Inflamm.

[6] Kok H, Barton K (2002). Uveitic glaucoma. Ophthalmol Clin N Am.

[7] Lloyd MJ, Mansberger SL, Fortune BA, Nguyen H, Torres R, Demirel S (2013). Features of optic disc progression in patients with ocular hypertension and early glaucoma. J Glaucoma.

[8] Giangiacomo A, Garway-Heath D, Caprioli J (2006). Diagnosing glaucoma progression: current practice and promising technologies. Curr Opin Ophthalmol.

[9] Quigley HA, Katz J, Derick RJ, Gilbert D, Sommer A (1992). An evaluation of optic disc and nerve fiber layer examinations in monitoring progression of early glaucoma damage. Ophthalmology.

[10] Leung CK, Cheung CYL, Weinreb RN, Qiu K, Liu S, Li H (2010). Evaluation of retinal nerve fiber layer progression in glaucoma: a study on optical coherence tomography guided progression analysis. Invest Ophthalmol Vis Sci.

[11] Grewal DS, Tanna AP (2013). Diagnosis of glaucoma and detection of glaucoma progression using spectral domain optical coherence tomography. Curr Opin Ophthalmol.

[12] Chauhan BC, Garway-Heath DF, Goñi FJ, Rossetti L, Bengtsson B, Viswanathan AC (2008). Practical recommendations for measuring rates of visual field change in glaucoma. Br J Ophthalmol.

[13] Folgar FA, de Moraes CGV, Prata TS, Teng CC, Tello C, Ritch R (2010). Glaucoma surgery decreases the rates of localized and global visual field progression. Am J Ophthalmol.

[14] Smith SD, Katz J, Quigley HA (1996). Analysis of progressive change in automated visual fields in glaucoma. Invest Ophthalmol Vis Sci.

[15] Advanced Glaucoma Intervention Study (1994). 2. Visual field test scoring and reliability. Ophthalmology.

[16] Iwao K, Inatani M, Seto T, Takihara Y, Ogata-Iwao M, Okinami S (2012). Long-term outcomes and prognostic factors for trabeculectomy with mitomycin C in eyes with uveitic glaucoma: a retrospective cohort study. J Glaucoma.

[17] Jabs DA, Nussenblatt RB, Rosenbaum JT (2005). Standardization of uveitis nomenclature for reporting clinical data. Results of the First International Workshop. Am J Ophthalmol.

[18] Lange C, Feltgen N, Junker B, Schulze-Bonsel K, Bach M (2009). Resolving the clinical acuity categories ‘hand motion’ and ‘counting fingers’ using the Freiburg Visual Acuity Test (FrACT). Graefes Arch Clin Exp Ophthalmol.

[19] Schulze-Bonsel K, Feltgen N, Burau H, Hansen L, Bach M (2006). Visual acuities ‘Hand motion’ and ‘Counting fingers’ can be quantified with the Freiburg Visual Acuity Test. Invest Ophthalmol Vis Sci.

[20] Edmunds B, Thompson JR, Salmon JF, Wormald RP (2001). The National Survey of Trabeculectomy. II. Variations in operative technique and outcome. Eye.

[21] Chawla A, Mercieca K, Fenerty C, Jones NP (2012). Outcomes and complications of trabeculectomy enhanced with 5-fluorouracil in adults with glaucoma secondary to uveitis. J Glaucoma.

[22] Foster PJ, Buhrmann R, Quigley HA, Johnson GJ (2002). The definition and classification of glaucoma in prevalence surveys. Br J Ophthalmol.

[23] Fitzke FW, Crabb DP, McNaught AI, Edgar DF, Hitchings RA (1995). Image processing of computerised visual field data. Br J Ophthalmol.

[24] Viswanathan AC, Fitzke FW, Hitchings RA (1997). Early detection of visual field progression in glaucoma: a comparison of PROGRESSOR and STATPAC 2. Br J Ophthalmol.

[25] Bhandari A, Crabb DP, Poinoosawmy D, Fitzke FW, Hitchings RA, Noureddin BN (1997). Effect of surgery on visual field progression in normal-tension glaucoma. Ophthalmology.

[26] Hong S, Seong GJ, Hong YJ (2007). Long-term intraocular pressure fluctuation and progressive visual field deterioration in patients with glaucoma and low intraocular pressures after a triple procedure. Arch Ophthalmol.

[27] Caprioli J, Coleman AL (2008). Intraocular pressure fluctuation a risk factor for visual field progression at low intraocular pressures in the advanced glaucoma intervention study. Ophthalmology.

[28] Broadway DC, Chang LP (2001). Trabeculectomy, risk factors for failure and the preoperative state of the conjunctiva. J Glaucoma.

[29] Ceballos EM, Parrish RK, Schiffman JC (2002). Outcome of Baerveldt glaucoma drainage implants for the treatment of uveitic glaucoma. Ophthalmology.

[30] Joshi AB, Parrish RK, Feuer WF (2005). 2002 survey of the American Glaucoma Society: practice preferences for glaucoma surgery and antifibrotic use. J Glaucoma.

[31] Chen PP, Yamamoto T, Sawada A, Parrish RK, Kitazawa Y (1997). Use of antifibrosis agents and glaucoma drainage devices in the American and Japanese Glaucoma Societies. J Glaucoma.

[32] Nguyen QH (2009). Primary surgical management refractory glaucoma: tubes as initial surgery. Curr Opin Ophthalmol.

[33] Kaburaki T, Koshino T, Kawashima H, Numaga J, Tomidokoro A, Shirato S (2009). Initial trabeculectomy with mitomycin C in eyes with uveitic glaucoma with inactive uveitis. Eye.

[34] Heijl A, Leske MC, Bengtsson B, Hyman L, Bengtsson B, Hussein M (2002). Reduction of intraocular pressure and glaucoma progression: results from the Early Manifest Glaucoma Trial. Arch Ophthalmol.

[35] Lam DSC, Fan DSP, Ng JSK, Yu CBO, Wong CY, Cheung AYK (2005). Ocular hypertensive and anti-inflammatory responses to different dosages of topical dexamethasone in children: a randomized trial. Clin Exp Ophthalmol.

